# Supplementary material for: Switchable Topological Polar Textures in Freestanding Ultrathin Ferroelectric Oxides
Source: Nano Lett. 2026 Apr 8;26(15):5258–65. doi: 10.1021/acs.nanolett.6c00763 (PMC13120705; doi:10.1021/acs.nanolett.6c00763)
Supplement: Supplementary file 1 [file nl6c00763_si_001.pdf]

Supporting Information for Switchable Topological Polar Textures in Freestanding Ultrathin Ferroelectric Oxides

Franco N. Di Rino* and Tim Verhagen*

Institute of Physics, Czech Academy of Sciences, Prague 8 182 00, Czech Republic

E-mail: dirino@fzu.cz; verhagen@fzu.cz

S1 Topological analysis

To characterize the chiral bubble–domain structure, we computed the vorticity $\boldsymbol{\omega}$ of the polarization field $\boldsymbol{P}$ (1),

$$\boldsymbol{\omega} = \nabla \times \boldsymbol{P}, \tag{S.1}$$

and identified helical cores by thresholding regions with large vorticity magnitude.

Streamlines were generated in ParaView (2) by interpolating the discrete polarization field, providing a qualitative visualization of how the polarization organizes into continuous toroidal loops. This representation highlights the three-dimensional arrangement of the helical cores, which periodically migrate between the top and bottom film surfaces. The analysis is intended as a qualitative tool to reveal the underlying topology rather than as a quantitative reconstruction of the vector field.

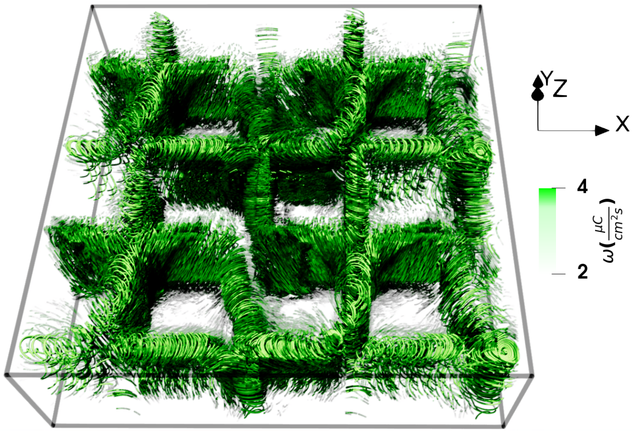

Figure S1: Thresholded vorticity regions. Streamlines highlight the local polarization flow and reveal how the core axes gradually tilt and bend, eventually tending to close into loop-like structures.

S2 Temperature evolution

The temperature evolution of selected properties for a representative $N_z = 4$ configuration is shown in Figure S2. A similar qualitative behavior is observed for all systems with $3 \leq N_z < 6$. At low temperatures, the in-plane polarization dominates and persists up to approximately 135 K, where the polarization magnitude vanishes, as shown in Figure S2a. Above this temperature, the system progressively enters a paraelectric-like regime, with the net polarization of all three components approaching zero and becoming increasingly governed by thermal fluctuations. This temperature also marks a crossover in the lattice parameters (Figure S2b), where the unit cells evolve from an orthorhombic-like to a tetragonal-like distortion. All quantities were obtained by averaging the polarization and lattice parameters over the entire simulation cell at each temperature.

S3 Structural analysis

To complement the topological analysis in the main text, a simple polarization-based criterion is used to assign the local structural character of each unit cell throughout the simulations. The thresholds to distinguish different polarization orientations are chosen to give a clear

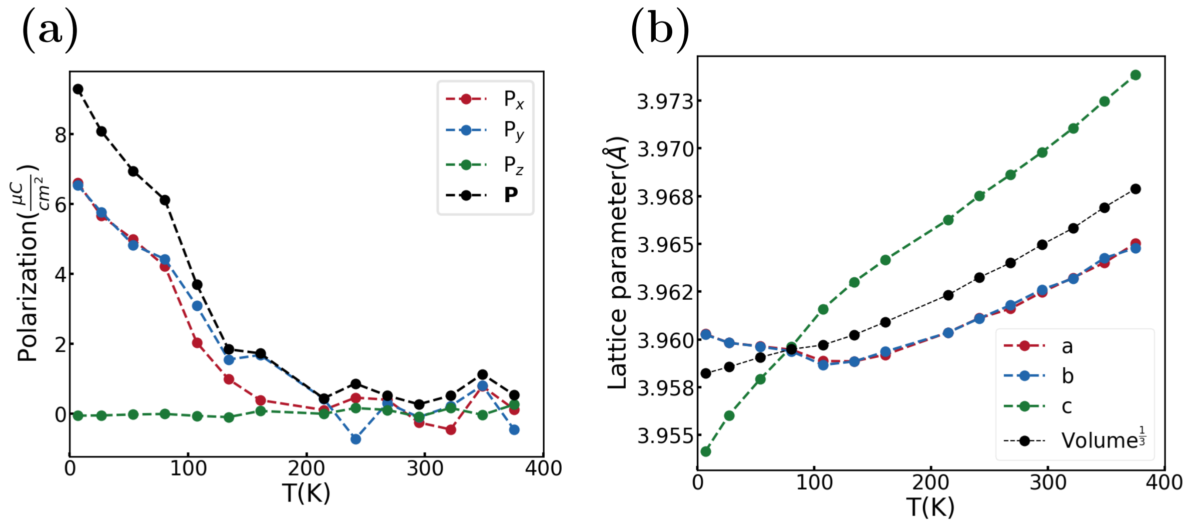

Figure S2: Temperature dependence of the average squared polarization (a) and the average lattice parameters (b) of a configuration with $N_z = 4$.

and physically reasonable separation. Although more sophisticated schemes exist (3), this minimal approach is sufficient to capture the structural trends relevant here.

All simulated free-standing BTO layers with thickness $N_z \geq 6$ display the same qualitative behavior discussed in the manuscript. Figure S3 shows the $N_z = 12$ system as a representative example, illustrating how this polarization-based analysis complements the topological characterization of the emergent textures.

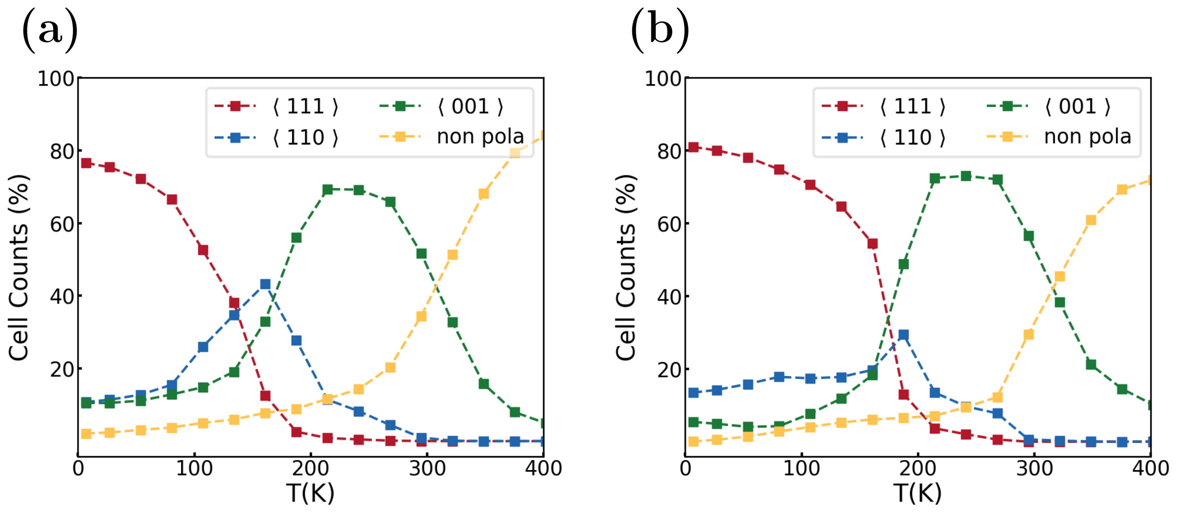

Figure S3: Exemplary $N_z = 12$ system structural evolution. (a) Starting from a chiral bubble state, the system follows the bulk-like rhombohedral–orthorhombic–tetragonal–paraelectric sequence under confinement. (b) Starting from wave-helix state, the orthorhombic phase does not clearly emerge.

S4 Flux-closure domains: Vortex-Antivortex textures

The field-induced reorganization in ultrathin layers ($3 \leq N_z < 6$) results in the formation of robust flux-closure patterns, as illustrated in Figure S4. This top-view map of the local polarization reveals a periodic arrangement of vortices and antivortices.

While the in-plane projection of these textures may qualitatively resemble the cross-sections of the chiral bubble states discussed in the main text, their three-dimensional description is fundamentally different. In the chiral bubble phase (see Figure 2c of the manuscript), the polarization exhibits a strong out-of-plane component (P_z), leading to the formation of asymmetric domains with a well-defined vertical orientation. In contrast, the flux-closure states shown here are characterized by a polarization vector that remains strictly confined to the layer plane, with a negligible out-of-plane contribution. This distinction confirms the essentially two-dimensional nature of the vortex-antivortex network in the ultrathin regime.

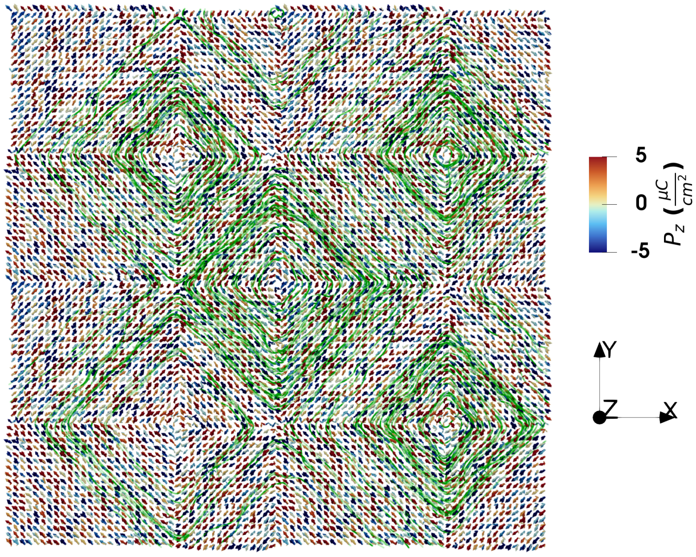

Figure S4: Top view of the polarization pattern in the ultrathin limit. The vector field displays a periodic array of vortices and antivortices. Streamlines serve as a guide to the eye to highlight the flux-closure nature of the domains, which lack an out-of-plane polarization component.

References

(1) Lukyanchuk, I. A.; Razumnaya, A. G.; Kondovych, S.; Tikhonov, Y. A.; Khesin, B.; Vinokur, V. M. Topological foundations of ferroelectricity. *Phys. Rep.* **2025**, *1110*, 1–56, DOI: 10.1016/j.physrep.2025.01.002.

(2) Ahrens, J.; Geveci, B.; Law, C. In *Visualization Handbook*; Johnson, C. R., Hansen, C. D., Eds.; Elsevier, 2005.

(3) Sepliarsky, M.; Aquistapace, F.; Di Rino, F.; Machado, R.; Stachiotti, M. G. Local-phase framework for the $BaTi_{1-x}Zr_xO_3$ phase diagram: From ferroelectricity to dipolar glass. *Phys. Rev. B* **2025**, *112*, 214105, DOI: 10.1103/kz7p-t99d.
